# My child cannot breathe while sleeping: a report of three cases and review

**DOI:** 10.1186/s12887-017-0922-9

**Published:** 2017-07-18

**Authors:** Won Hee Seo, Minkyu Park, So-Hee Eun, Seonkyeong Rhie, Dae Jin Song, Kyu-Young Chae

**Affiliations:** 1 Department of Pediatrics, Korea University College of Medicine, Seoul, Korea; 2 Department of Pediatrics, Hanil Medical Center, Seoul, Korea; 3 Department of Pediatrics, CHA Bundang Medical Center, CHA University, 59 Yatapro, Seongnam, 13496 GyungGi Province Korea

**Keywords:** Breath-holding spell, Gastroesophageal reflux, Laryngospasm, Nocturnal seizure, Polysomnography, Sleep

## Abstract

**Background:**

Sudden breath-holding episodes during sleep in young children are potentially related to sudden infant death syndrome and other life-threatening events. Additionally, these episodes can negatively affect child’s growth and development.

**Case presentation:**

Here, we present 3 cases of preschool children with similar paroxysmal nocturnal waking events associated with choking that had different etiologies (nocturnal frontal lobe epilepsy, nocturnal gastroesophageal reflux disease, and parasomnia, respectively).

**Conclusions:**

It is important to take into consideration the fact that breath spells during sleep can occur as a rare manifestation of parasomnia due to gastroesophageal reflux or as a symptom of nocturnal frontal lobe epilepsy. Full video electroencephalography, polysomnography, and simultaneous gastric pH monitoring should be used for the differential diagnosis of sleep-related disorders, such as breath spells, in children.

## Background

Sudden breath-holding and cyanotic events during sleep are known to cause health complications in young children. These episodes can be frightening and cause intense hypnophobia. Frequent nighttime awakening can also have detrimental effects on child’s growth and development, as well as adversely affect the health of the entire family. The burden of caring for affected children can decrease family’s productivity, increase the incidence of family conflict-related stress, and even lead to maternal depression [1]. Thus, the early diagnosis and management of nocturnal breath-holding episodes are critical for maintaining the quality of life of both the affected child and family.

A variety of conditions can cause nocturnal waking episodes in childhood. Frequent nocturnal insomnias can be related to an acute medical condition, such as otitis media, sleep-onset association disorders caused by a lack of sleep training, such as childhood behavioral insomnia, primary sleep disorders, such as sleep-related breath disorder, or partial arousal parasomnias, such as night terrors and confusional arousals [2, 3].

Paroxysmal nocturnal events are commonly misdiagnosed as epilepsy. However, nocturnal epilepsy and parasomnias are distinct causes of breath-holding during sleep that should be differentiated [4, 5]. In addition, laryngospasm is a rare cause of abrupt breath-holding during sleep that is caused by involuntary muscular contraction of the laryngeal cords [6, 7]. Extraesophageal manifestations of the gastroesophageal reflux disease (GERD) are a known cause of laryngospasm.

Polysomnography (PSG) is the standard method for the diagnosis of sleep disorders. Alternatively, video electroencephalography (EEG) can provide useful diagnostic information in individuals with a history of prominent motor activity during sleep [8, 9]. However, the isolated use of these tools is often insufficient for the correct diagnosis of nocturnal events.

In this report, we present three cases of preschool-aged children who had similar symptoms (i.e., multiple abrupt waking with choking episodes every night), but received different diagnoses based on PSG with full-montage of video-EEG, as well as 24-h esophageal pH monitoring.

## Case presentation

### Case 1

A 4-year-old girl was admitted to our clinic with a 2 years history of choking episodes during sleep that occurred 4–5 times almost every night. The patient had no familial history of epilepsy or allergic disease, and her weight and height were within the normal range according to the Korean growth charts.

During the initial evaluation performed 2 years before, brain magnetic resonance imaging (MRI) and standard PSG revealed no abnormalities. Because the EEG revealed few focal epileptiform discharges in the left frontal area, the patient was prescribed an antiepileptic medication; however, her nocturnal spells persisted.

Upon the admission, we performed PSG with video-EEG. Five choking episodes were observed on the video; the patient awakened suddenly, sat up, and experienced episodes of choking, air hunger, agitation, and intense fear; in two episodes, with the patient showed gagging gestures just after awakening. During these periods, PSG recording and diagnostic parameters showed that the patient was in stage 3 of non-rapid eye movement (NREM) sleep and experienced obstructive or mixed-type apnea, followed by arousal and awakening with dyspnea and tachycardia. The peripheral oxygen saturation (Sp0_2_) decreased to 83%. During these episodes, the patient’s parents performed back percussion and provided reassurance. Episodes typically resolved within 1–3 min and the patient fell asleep again after each event.

**Fig. 1:**
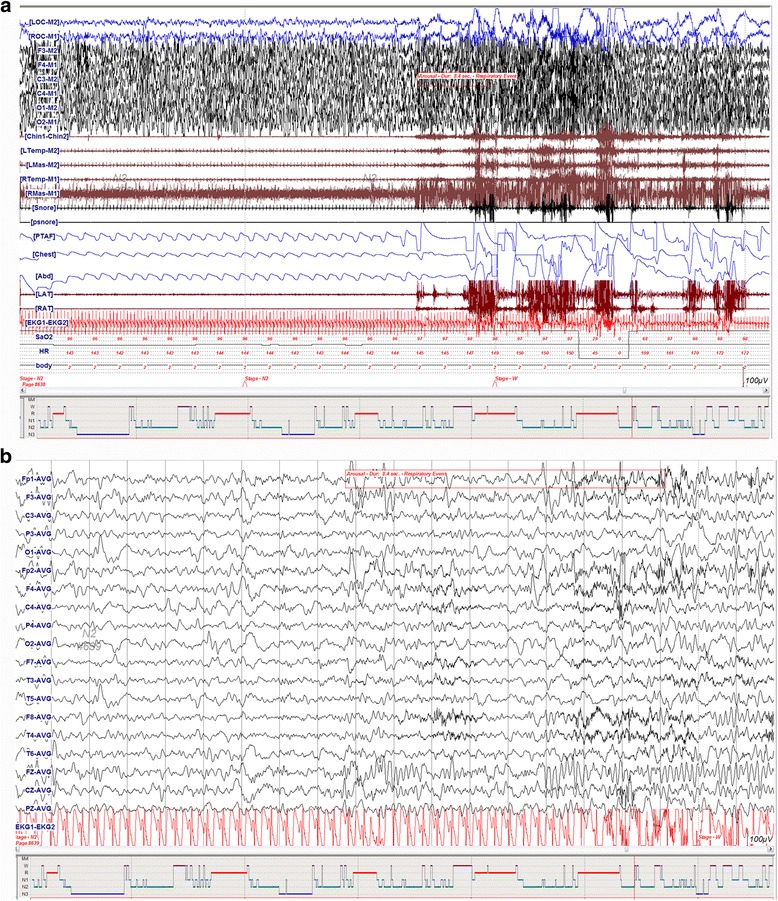
**a** Polysomnographic recording during sleep. The patient presented suddeneye opening followed by breath-holding with or without agitation and nausea; **b** Full-montage EEG showingsharp wave discharges intermixed with rhythmic 6–7 Hz activities in the frontal polar and anterior midlineregions during the nocturnal events

We attempted a third diagnostic session of PSG with full-montage of video-EEG and simultaneous 24-h gastric pH monitoring in order to exclude the possibility of GERD. Out of 6 choking episodes, 4 were associated with low-amplitude 13–14 Hz fast activities in the right or left frontal areas (Fz, F3, F4) that evolved into medium-to-high-amplitude bi-frontal sharp wave discharges intermixed with 6–7 Hz activities in the posterior cerebral region. Accompanying obscuring muscle artifacts were observed and presented as sudden eye opening and right arm tonicity, followed by breath-holding with or without agitation and gagging (Fig. 1a, b). Several low pH (< 4) events were observed on simultaneous 24-h pH monitoring; however, these episodes did not coincide with the nocturnal breath-holding spells. The patient was diagnosed with frontal lobe epilepsy and experienced complete resolution of her nocturnal symptoms after intravenous administration of 20 mg/kg of sodium valproate, and then, the patient was prescribed oral valproate (Depakoke15mg/kg/day, twice a day). 1 month later, the patient had added topiramate. On follow up until 1 year after, it had been reported that the patient’s symptoms were well controlled with only sporadic manifestation of nocturnal episodes.

### Case 2

A 4-year-old girl was admitted complaining of 1–3 nightly episodes of sudden nocturnal waking with choking or coughing that had occurred over the previous 40 days. The patient’s body weight (13 kg) and height (91 cm) were in the 3rd percentile for Korean girls according to the Korean growth charts. The patient had no history of medical or neurological problems from birth, and no familial history of epilepsy or allergic disease.

The patient had sudden awakening events accompanied by intense fear due to difficulty breathing during sleep. After each episode, the patient was unable to speak or cry, but these symptoms resolved in 1–3 min with back percussion and reassurance from her parents. Subsequently, the child would cry and return to sleep.

**Fig. 2:**
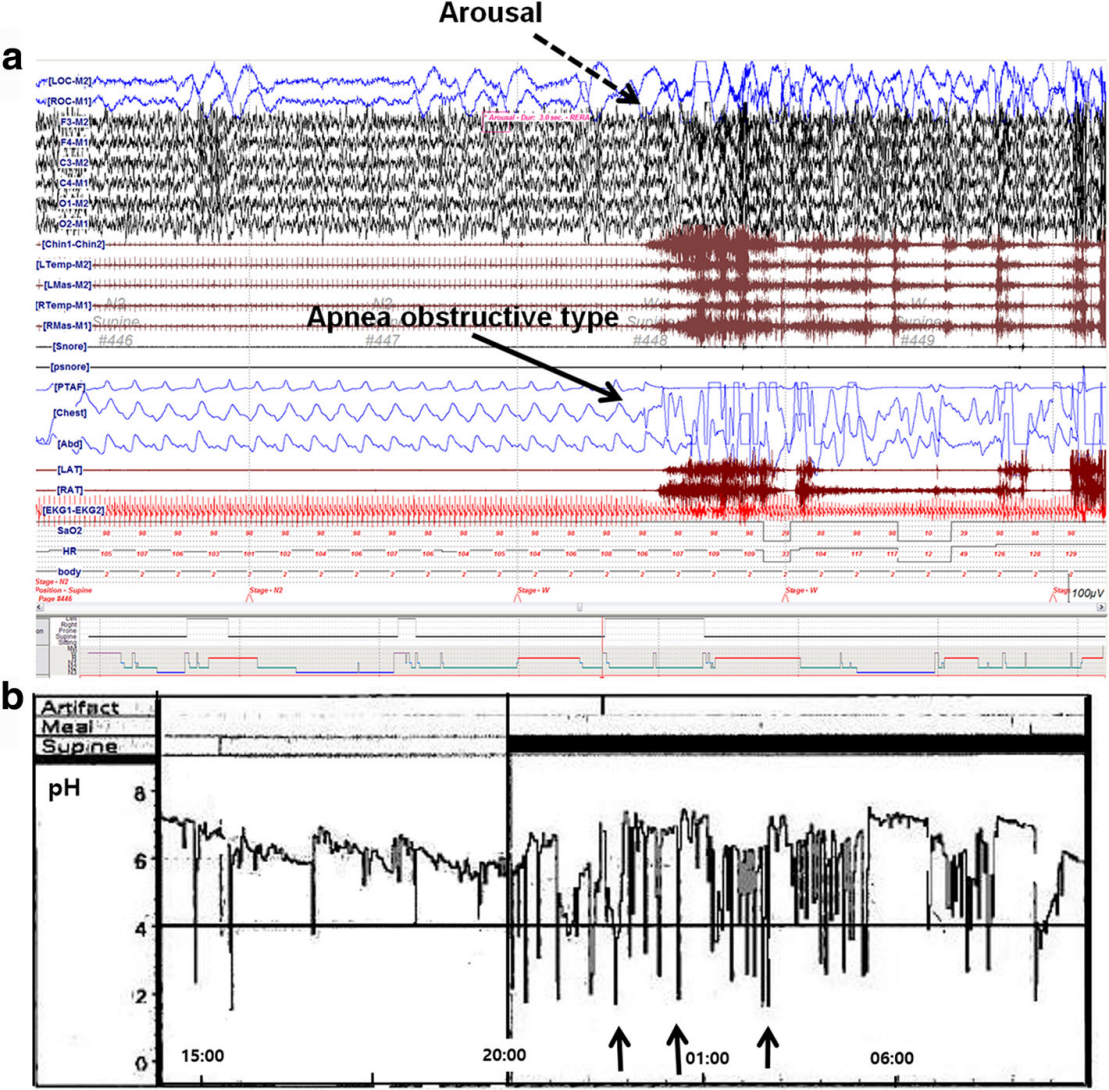
**a** Polysomnographic recording during nocturnal events. The patient was in N3 sleep and presented apnea after arousal; **b** Results of 24-h pH monitoring showing nocturnal events (*black arrows*) with low pH (<4), which wereindicative of gastroesophageal reflux

No abnormalities were detected by respiratory tests, neurological tests, full-montage EEG, or brain MRI. Upon physical examination, the patient presented adenoid hypertrophy and it was noted that she had snoring episodes 1–2 times per week. Her overnight PSG revealed repetitive choking and inspiratory stridor followed by sudden cortical arousal with obstructive apnea (Fig. 2a). Simultaneous 24-h gastric pH monitoring revealed significant gastroesophageal reflux (GER) during nocturnal events (Fig. 2b). Erosive esophagitis was subsequently found by gastroscopy. The patient was diagnosed with nocturnal GERD and received anti-reflux therapy, including gastrokinetic agents and proton pump inhibitors (PPI) that completely resolved the symptoms within 2 weeks.

### Case 3

A 5-year-old girl was admitted with complaints of 2–3 nightly episodes of sudden waking and choking that occurred during the previous 5 days. The episodes usually occurred between 02:00–04:00 AM and were sometimes accompanied by coughing. The patient had no familial history of epilepsy or allergic disease.

The patient’s parents witnessed sudden awakening accompanied by intense fear due to difficult breathing during sleep. During some episodes, the patient complained of chest discomfort and dyspnea. Each episode lasted 5–6 min and resolved after back percussion and reassurance from the parents. After each episode, the patient returned to sleep and was unable to recall the episode the following morning.

No abnormalities were detected by respiratory tests, neurological tests, full-montage EEG, or brain MRI. Upon physical examination, the patient presented adenoid hypertrophy and it was noted that the patient had snoring episodes 3–4 times per week. During the overnight PSG, the patientawoke3 times due to choking and crying during NREM and REM sleep for periods of about 15 s each. During the episodes, Sp0_2_ decreased to 87%. No epileptiform discharges were noted. Gastroscopy results were normal; further, the patient experienced a worsening of her nocturnal symptoms during empirical anti-reflux medication. The patient was finally diagnosed with parasomnia and was prescribed clonazepam. All symptoms resolved completely within 10 days.

## Discussion

Here we described 3 cases of childhood nocturnal breath-holding episodes, each related to a different diagnosis. A major conclusion of this report is that the differential diagnosis for paroxysmal breath-holding spells during sleep should consider nocturnal seizures and sleep disorders, such as parasomnia, sleep-related laryngospasm associated with GERD, and obstructive sleep apnea.

Insufficient or lack of sleep is a common trigger factor for developing seizures and epileptiform discharges on EEG. Children with epilepsy commonly complain of sleep disturbances, including nighttime awakening, particularly when seizures are poorly controlled. [4] Nocturnal frontal lobe epilepsy and benign focal childhood epilepsy with centrotemporal spikes are a few more common epileptic syndromes that present with nocturnal seizures [5].

Nocturnal frontal lobe epilepsy is characterized by various motor behaviors during sleep, including pedaling movement of the limbs, dystonic posturing, and tremors after sudden arousal. [5] Because the interictal EEGs are usually normal in cases with nocturnal frontal lobe epilepsy, the differentiation of this disorder from partial arousal parasomnias or sleep-related movement disorders may be difficult when the nocturnal symptoms are nonspecific.

Benign focal childhood epilepsy with centrotemporal spikes is characterized by partial seizures with sensorimotor symptoms of the face and oropharynx, hemiconvulsions, and tonic-clonic seizures during NREM sleep [5]. Seizures usually occur on a nightly basis during sleep or upon awakening.

When nocturnal seizures are eliminated as a diagnostic possibility, the suggested differential diagnosis includes sleep-related choking syndrome or laryngospasm, which can be associated with GER during sleep. Although GER commonly occurs in infants and resolves with age, the persistence of nocturnal GERD is a known cause of sleep disturbances in children. Sleep-related choking syndrome or laryngospasm has been described in the diagnostic and coding manual of the American Sleep Disorder Association as a dramatic attack that produces breathing difficulties, awakening, stridor, apnea, fear, and agitation that typically resolves within 1–3 min [10].Reflux-induced apnea affects approximately 1% of infants and is related to airway closure or laryngospasm [6]. Recently, GER studies have revealed that weak acid reflux during the nighttime occurs more frequently than was previously considered and may trigger nocturnal episodes of laryngospasm [6, 7]. The extreme reported cases of laryngospasm related to GERD include Sandifer syndrome, sleep-related laryngospasm, refractory asthma, and idiopathic pulmonary fibrosis [6, 11, 12]. Sandifer syndrome is often misdiagnosed as epilepsy and is characterized by the head rotation and tonic posture. Manifestations of this syndrome typically occur in the postprandial period [11].

Despite the fact that the exact underlying mechanisms of GER-induced laryngospasm remain unknown, the overreaction of the laryngeal chemoreflex (LCR) has been suggested as a potential trigger in the laryngospasm pathophysiology. The LCR protects the subglottic airway from the liquid materials and the lungs from aspiration; this reflex consists of swallowing, coughing, and arousal aimed at limiting the duration of contact between the liquids and the laryngeal mucosa [13, 14]. However, in some infants, the laryngospasm triggered by LCR and associated with nocturnal symptoms, including central or mixed/obstructive apnea, oxygen desaturation, and bradycardia due to a vagal efferent component during the sleep, can be life-threatening. The LCR can be triggered by GER, bottle-feeding, or oral intake of medicine. Moreover, several physiological changes during sleep can induce GER, including prolonged esophageal acid contact time, decreased upper esophageal sphincter pressure, increased gastric acid secretion, decreased salivation, and decreased swallowing [15]. Several case reports have indicated improvement of the nocturnal symptoms of sleep-related laryngospasm after GERD treatment [16–18]. Given the fact that the acid-suppressing therapies and proton pump inhibitor treatments have been widely used for supra-esophageal manifestations of the infantile GERD and suspected silent GERD, these medications may be useful for the treatment of GER-induced laryngospasm in children [15]. In our second case, the evidence of acid reflux was detected via gastroscopy and PSG with simulataneous 24-h gastric pH monitoring. Subsequent treatment with anti-reflux therapy gradually decreased the frequency of nocturnal episodes, and the symptoms effectively disappeared after 2 months.

Parasomnia often manifests during the transition from wakefulness to sleep. Although the exact pathophysiology of parasomnia remains unclear, it is considered a multifactorial disease that is related to an interplay of genetic and environmental factors [19]. Sleep talking, sleep walking, and confusional arousals are common symptoms of parasomnia in childhood. Regular and adequate sleep routines, as well as good sleep hygiene are suggested for the management of parasomnia in young children. Medications, including benzodiazepines, are considered dangerous for these patients and are unadvised for the treatment of parasomnia [20]. Thus, care should be taken to differentiate the parasomnia from organic disease states, such as nocturnal seizures and GERD.

PSG is a standard method for the diagnosis of sleep disorders; however, the standard PSG alone does not allow the differentiation of nocturnal seizures from other sleep disorders. PSG with full-montage of video-EEG is superior to standard PSG for the evaluation of nocturnal spells, because of an increased capacity for the identification and localization of EEG abnormalities and the ability to correlate patient behavior with EEG and PSG data [8, 9]. Esophageal pH probe monitoring combined with cardio-respiratory monitoring using nasal airflow for the detection of obstructive apnea has been used to clarify temporal associations between the reflux and apnea. Moreover, a careful collection and examination of patient’s medical history is helpful for the diagnosis of sleep apnea (e.g., noting daytime nausea or vomiting when the child is in a supine or seated position after feeding, as signs of airway obstruction in tandem with these symptoms may indicate the involvement of GER). Thus, 24-h pH monitoring with simultaneous PSG with full-montage of video-EEG is an ideal modality for the diagnosis of nocturnal breath-holding events, especially in young patients.

## Conclusions

In conclusion, breath-holding episodes during sleep should be differentiated from nocturnal seizures and other sleep disorders. When diagnosing nocturnal episodes, it is important to take into consideration the fact that sleep-related choking syndrome and laryngospasm associated with GER may appear in the absence of other GERD symptoms. PSG is an essential tool for the evaluation of sleep abnormalities; however, PSG alone is not useful for the differentiation of nocturnal seizures and other sleep disorders in children. Therefore, PSG with full-montage video-EEG and simultaneous 24-h pH monitoring are important adjunct tools for the diagnosis of respiratory symptoms, such as breath spells, choking, and sudden coughing during sleep.

## Abbreviations

EEG: Electroencephalography;
GER: Gastroesophageal reflux
GERD: Gastroesophageal reflux disease
LCR: Laryngeal chemoreflex
MRI: Magnetic resonance imaging
NREM: Non-rapid eye movement
PSG: Polysomnography
REM: Rapid eye movement
